# Asymmetrical canina meiosis is accompanied by the expansion of a pericentromeric satellite in non-recombining univalent chromosomes in the genus *Rosa*

**DOI:** 10.1093/aob/mcaa028

**Published:** 2020-02-25

**Authors:** Jana Lunerová, Veit Herklotz, Melanie Laudien, Radka Vozárová, Marco Groth, Aleš Kovařík, Christiane M Ritz

**Affiliations:** 1 Department of Molecular Epigenetics, Institute of Biophysics, Czech Academy of Sciences, Brno, Czech Republic; 2 Department of Botany, Senckenberg Museum of Natural History Görlitz, Görlitz, Germany; 3 Technical University Dresden, International Institute Zittau (IHI), Chair of Biodiversity of Higher Plants, Zittau, Germany; 4 Masaryk University, Faculty of Science, Brno, Czech Republic; 5 Leibniz Institute on Ageing – Fritz Lipmann Institute, Jena, Germany

**Keywords:** *Rosa*, dogroses, polyploidy, meiosis, satellite DNA, chromosome evolution, repeatome, genetic recombination

## Abstract

**Background and Aims:**

Despite their abundant odd-ploidy (2*n* = 5*x *= 35), dogroses (*Rosa* sect. *Caninae*) are capable of sexual reproduction due to their unique meiosis. During canina meiosis, two sets of chromosomes form bivalents and are transmitted by male and female gametes, whereas the remaining chromosomes form univalents and are exclusively transmitted by the egg cells. Thus, the evolution of chromosomes is expected to be driven by their behaviour during meiosis.

**Methods:**

To gain insight into differential chromosome evolution, fluorescence *in situ* hybridization was conducted for mitotic and meiotic chromosomes in four dogroses (two subsections) using satellite and ribosomal DNA probes. By exploiting high-throughput sequencing data, we determined the abundance and diversity of the satellite repeats in the genus *Rosa* by analysing 20 pentaploid, tetraploid and diploid species in total.

**Key Results:**

A pericentromeric satellite repeat, CANR4, was found in all members of the genus *Rosa*, including the basal subgenera *Hulthemia* and *Hesperhodos*. The satellite was distributed across multiple chromosomes (5–20 sites per mitotic cell), and its genomic abundance was higher in pentaploid dogroses (2.3 %) than in non-dogrose species (1.3 %). In dogrose meiosis, univalent chromosomes were markedly enriched in CANR4 repeats based on both the number and the intensity of the signals compared to bivalent-forming chromosomes. Single-nucleotide polymorphisms and cluster analysis revealed high intragenomic homogeneity of the satellite in dogrose genomes.

**Conclusions:**

The CANR4 satellite arose early in the evolution of the genus *Rosa*. Its high content and extraordinary homogeneity in dogrose genomes is explained by its recent amplification in non-recombining chromosomes. We hypothesize that satellite DNA expansion may contribute to the divergence of univalent chromosomes in *Rosa* species with non-symmetrical meiosis.

## INTRODUCTION

Polyploidy is a ubiquitous phenomenon in plants; whole genome duplications have been detected in all major lineages (Van de Peer *et al.*, 2017), and approximately one-quarter of extant plant species are polyploid (Barker *et al.*, 2016). Whole genome duplications arise from many different molecular mechanisms and lead to immediate and long-term transcriptomic, genomic and chromosomal reorganization (Alix *et al.* 2017). As a long-term effect, this reorganization results in genome downsizing and diploidization (Wendel, 2015). An immediate challenge that faces newly arisen polyploids is the maintenance and stability of sexual reproduction based on an effectively working meiosis. This is usually more easily gained by allopolyploids than by autopolyploids because the first originated by hybridization, and their homeologous genomes show often selective chromosome pairing (Ramsey and Schemske, 1998; Grandont *et al.*, 2014). In contrast, some polyploids, especially those with odd numbers of chromosomes, reproduce by apomixis, omitting meiosis completely (e.g. *Rubus*, *Taraxacum*). Very few plant groups have independently evolved intermediate hemisexual mechanisms of meiotic recombination, in which there is differentiation between recombining (bivalent-forming) and apomictically inherited (univalent-forming) chromosomes, such as *Rosa* sect. *Caninae* (Täckholm, 1922), *Leucopogon* (Epacridaceae; Grant, 1971) and *Onosma* (Boraginaceae; Teppner, 1971; Kolarčik *et al.*, 2018).

Section *Caninae* of the genus *Rosa* represents a highly successful group of wild roses called dogroses (~60 species in total) that occupy wide areas in northern and central Europe (Wissemann, 2003; Kurrto *et al.*, 2004; Koopman *et al.*, 2008). One species, *Rosa rubiginosa*, is an invasive neophyte in South America, South Africa and Australia (Hirsch *et al.*, 2011). Dogroses are exclusively polyploids; most species are pentaploid (2*n* = 5*x* = 35), and tetraploids, hexaploids and heptaploids (2*n* = 4*x*, 6*x*, 7*x* = 28, 42, 49) are less common (Wissemann, 2003; Pachl, 2011; Herklotz and Ritz, 2017). To cope with the abundant odd-ploidy, sect. *Caninae* evolved a peculiar reproductive system based on a type of asymmetrical meiosis called ‘canina meiosis’, which was described almost a century ago (Täckholm, 1920, 1922; Blackburn and Harrison, 1921; Blackburn, 1925). Regardless of ploidy level, only 14 chromosomes pair in meiosis (seven bivalents) and are regularly distributed to the male and female gametes, while the remaining chromosomes are transmitted as univalents via the egg cell (Täckholm, 1922). Thus, male and female gametes contribute unequally to the chromosome complement of the zygote, resulting in a matroclinal inheritance of genetic material. These early cytogenetic observations are supported by molecular studies involving the transmission of microsatellite alleles and randomly amplified polymorphic DNA (RAPD) markers, deviations from standard embryo/endosperm genome size ratios (Kolarčik *et al*., 2018) and sequences of nuclear ribosomal internal transcribed spacer regions (nrITS) in interspecific hybrids (Werlemark and Nybom, 2001; Wissemann, 2002; Nybom *et al.*, 2004, 2006; Ritz and Wissemann, 2011; Khaitová *et al.*, 2014). Despite this unique meiotic mechanism, the origin of sect. *Caninae* is not fully understood. Studies based on nrITS data have shown that dogroses originated by multiple hybridization, incorporating Caninae-specific ribotypes (ITS variants) and ribotypes that are also found in other sections of *Rosa* with regular meiosis (Wissemann, 1999; Ritz *et al.*, 2005). The Caninae-specific ribotypes mark the bivalent-forming genomes (Kovařík *et al.*, 2008) but are differentiated between subsections of dogroses; the Canina type was found in subsect. *Caninae* and the Rubiginosa type in subsect. *Rubigineae* (Herklotz *et al.*, 2018). Consistent with this, phylogenies based on plastid data suggest a polyphyletic origin of dogroses in that the clade comprising glandular species (subsect. *Rubigineae*, *Vestitae*) is separated from the non-glandular species (subsect. *Caninae*) by intermingled species of sect. *Gallicanae* and sect. *Synstylae* with regular meiosis (Wissemann and Ritz, 2005; Bruneau *et al.*, 2007; Fougere-Danezan *et al.*, 2015).

Although cytogenetic methods offer an excellent means of studying the effects of preferential chromosome pairing in dogroses, our knowledge of chromosome structure in dogroses is rather limited. Reports involving *in situ* hybridization on chromosomes of dogroses are particularly scarce, mainly due to the polyploid nature of the species and to difficulties in preparing chromosomes from woody plants. In addition, roses have a small genome size [the diploid genome size is 0.83–1.30 pg/2C (Roberts *et al.*, 2009)], small chromosomes (Kirov *et al.*, 2014; Hwang *et al.*, 2017), a low mitotic index in roots and shoots, and weak root development (Ma *et al.*, 1996). Attempts to apply the C-banding technique to mitotic chromosomes of roses resulted in poor resolution of bands (Price *et al.*, 1981). Nevertheless, information about the number and positions of rDNA sites in several dogrose species has been obtained. In general, there is a single 35S rDNA locus and a single 5S rDNA locus per haploid set [Ma *et al.*, 1997 and http://www.plantrdnadatabase.com/ (Garcia *et al.*, 2012)], and these are often located on the same chromosome (Ding *et al.*, 2016). Exceptionally, more than one 5S rDNA site per monoploid set was reported in both diploids (Kirov *et al.*, 2016) and polyploids (Herklotz *et al.*, 2018).

In general, the identification of effective cytogenetic markers is an important step in answering many biological questions. DNA satellites have been widely used by plant cytogeneticists as convenient chromosome markers due to their high intragenomic homogeneity, discrete hybridization signals on chromosomes and relative ease of isolation. Satellite DNA consists of numerous tandemly arranged repeats that are non-coding, late-replicating in S-phase, and mostly located in constitutive, non-transcribed heterochromatin (Plohl *et al.*, 2008; Garrido-Ramos, 2017). Their molecular properties, such as monomer unit divergence, copy number variation and changes in position on chromosomes, together with their overall rapid evolution have been used to discriminate between subgenomes in interspecific hybrids and allopolyploids (Renny-Byfield *et al.*, 2012; Zhang *et al.*, 2014; Kirov *et al.*, 2017; for a review, see Hemleben *et al.*, 2007).

In this study, we asked the following evolutionary questions: (1) What is the chromosome location and homogeneity of satellite repeats in dogroses? (2) Can univalent- and bivalent-forming chromosomes in dogroses be distinguished based on satellite markers? (3) What is the divergence and abundance of satellite repeats across the genus *Rosa*? To answer these questions, we combined conventional molecular methods with genomic approaches. We analysed repeatomes in 5*x* dogroses (six species) and compared the data with data from 14 2*x* and two 4*x* rose species with regular meiosis covering the major sections of *Rosa*. We identified an abundant satellite repeat, CANR4, in dogroses; this satellite, which is present in varying amounts across the genus *Rosa*, was used as a probe for fluorescence *in situ* hybridization (FISH) analyses in mitotic and meiotic metaphases.

## MATERIAL AND METHODS

### Plant material

We investigated a natural mixed stand of dogroses (WGS84: 51.1732°N, 14.6271°E, Weißenberg, Germany) comprising several species of subsections *Rubigineae* and *Caninae*. The herbarium vouchers for these species are given in Herklotz and Ritz (2014, 2018). The 5*x Rosa canina* CZ was also collected in a single locality (WGS84: 49.2310°N, 16.5957°E, Brno, Czech Republic). *Rosa gallica* was sampled from a natural stand (WGS84: 49.8396°N, 9.8926°E, Veitshöchsheim, Germany). *Rosa rubiginosa* and *R. sherardii* (both 5*x* species) were gifts of H. Nybom (University of Agricultural Science, Balsgård, Sweden); *R. rubiginosa* has been further cultivated in the Academy campus garden (49.2207°N, 16.5809°E, Brno-Žabovřesky) since 2008. The remaining rose species sampled by ourselves were obtained from the wild rose collection hosted by the Botanical Garden of the University Würzburg, Germany, and established by V. Wissemann (Justus-Liebig-Universität Gießen, Germany) and from the Forstpark Tharandt (Technical University Dresden). One accession of *Fragaria moschata* Weston (WGS84: 51.1942°N, 13.5543°E, Oberau, Germany) was used as an outgroup reference in the Southern blot experiment. In addition, we used sequence information available from the European Nucleotide Archives (ENA) to investigate rose accessions. The analytical methods used in this study are summarized in Table 1.

**Table 1.** List of *Rosa* species, type of analysis and read archive accessions used in this study

Subgenus^a^	Section/subsection	Species	Ploidy	Source^b^	Sampling details	Methods applied	Read archive (run accession)
*Hesperhodos* Cockerell		*R. minutifolia* Engelm.	2*x*	ENA		HTS	SRR7077023^c^
*Hulthemia* (Dumort.) Focke		*R. persica* Juss.	2*x*	ENA		HTS	SRR7077021^c^
*Rosa*	*Caninae* (DC.) Ser./*Caninae*	*R. canina* L. (CZ_1)	5*x*	CZ, Brno		HTS, FISH	SRR8265808^d^
*Rosa*	*Caninae*/*Caninae*	*R. canina* L. (CZ_2)	5*x*	CZ, Brno		FISH	–
*Rosa*	*Caninae*/*Caninae*	*R. canina* L. (CZ_3)	5*x*	CZ, Brno		FISH	–
*Rosa*	*Caninae*/*Caninae*	*R. canina* L. (DE_S27b)	5*x*	DE, Weißenberg	GLM12396	HTS, FISH, SB	ERR1662939^e^
*Rosa*	*Caninae*/*Caninae*	*R. corymbifera* Borkh. (DE_S2)*	5*x*	DE, Weißenberg	Herklotz and Ritz (2014) / GLM49579	HTS	SRR8265810^d^
*Rosa*	*Caninae*/*Caninae*	*R. dumalis* Bechst.	5*x*	DE, Weißenberg	Herklotz and Ritz (2014) / GLM49831	HTS, SB	ERR1662941^e^
*Rosa*	*Caninae*/*Rubiginae* Christ	*R. inodora* Fr.	5*x*	DE, Weißenberg	Herklotz and Ritz (2014) /GLM4959	HTS, FISH, SB	ERR1662940^e^
*Rosa*	*Caninae*/*Vestitae* Christ	*R. sherardii* Davies	5*x*	SW, Balsgaard	accession 1402	HTS	SRR10402273^d^
*Rosa*	*Caninae*/*Rubiginae*	*R. rubiginosa* L.	5*x*	SW, Balsgaard	Kovařík *et al* (2008) accession 1408	HTS, FISH	SRR10402274^d^
*Rosa*	*Caninae*/*Rubiginae*	*R. agrestis* Savi	6*x*	DE, Weißenberg	Herklotz and Ritz (2014)/GLM4959	SB	–
*Rosa*	*Gallicanae* (DC.) Ser.	*R. gallica* L.	4*x*	DE, BG Würzburg	GLM-172058	HTS, FISH	SRR8422952^e^
*Rosa*	*Indicae* Thory	*R. chinensis* Jacq.	2*x*	ENA		HTS	SRR7077020^c^
*Rosa*	*Indicae*	*R. gigantea* Crép.	2*x*	ENA		HTS	SRR6175516^c^
*Rosa*	*Laevigatae* Thory	*R. laevigata* Michx.	2*x*	ENA		HTS	SRR7077018^c^
*Rosa*	*Rosa*	*R. californica* Cham. & Schltdl.	2*x*	BG Tharandt		SB	
*Rosa*	*Rosa*	*R. carolina* L.	2*x*	BG Tharandt		SB	
*Rosa*	*Rosa*	*R. majalis* Herrm.	2*x*	DE, BG Würzburg	GLM-172056	HTS, FISH, SB	SRR8422953^d^
*Rosa*	*Rosa*	*R. pendulina* L.	2*x*	BG Tharandt		SB	
*Rosa*	*Rosa*	*R. rugosa* Thunb.	2*x*	ENA		HTS, SB	SRR7077019^c^
*Rosa*	*Rosa*	*R. virginiana* Mill.	2*x*	BG Tharandt		SB	
*Rosa*	*Rosa*	*R. woodsia* Lindl.	2*x*	BG Tharandt		SB	
*Rosa*	*Pimpinellif.* (DC.) Ser.	*R. spinosissima* L.	4*x*	DE, BG Würzburg	GLM-172057	HTS, FISH	SRR8422951^d^
*Rosa*	*Pimpinellif.*	*R. xanthina* Lindl.	2*x*	ENA		HTS	SRR7077022^c^
*Rosa*	*Synstylae* DC.	*R. arvensis* Huds.	2*x*	DE, BG Würzburg	GLM-172055	HTS, FISH, SB	SRR8265809^d^
*Rosa*	*Synstylae*	*R. moschata* Herrm.	2*x*	ENA		HTS	SRR7077017^c^
*Rosa*	*Synstylae*	*R. multiflora* Thunb.	2*x*	ENA		HTS	SRR7077023^f^

Abbreviations: BG, Botanical Garden; *Pimpinellif.*, *Pimpinellifoliae*; FISH, fluorescence *in situ* hybridization; HTS, high-throughput sequencing; SB, Southern blot hybridization.

^a^Taxonomy is according to Wissemann (2003).

^b^CZ, Czech Republic; DE, Germany; SW, Sweden.

^c^European Nucleotide Archives (ENA) (Saint-Oyant *et al.*, 2018).

^d^This work (ENA).

^e^ENA sequence archive (Herklotz *et al.*, 2018).

^f^ENA (Nakamura *et al.*, 2018).

### DNA extraction and purification

Genomic DNA was isolated from silica-gel-dried leaflets. Because conventional methods of extraction often result in poor yields and strong contamination with polyphenolic compounds, the ATMAB protocol was used (Dumolin *et al.*, 1995).

### Cloning of the CANR4 satellite

Purified DNA from *R. canina* was digested with the *Rsa*I restriction enzyme (cleavage at the GTAC motif), resulting in a high-molecular-weight band that hybridized strongly with a genomic probe. The band was cut and the DNA digested as a whole in an agarose block with *Mbo*I (motif: GATC). The resulting fragments were isolated using a PCR clean-up kit (Macherey-Nagel, Düren, Germany). The DNA fragments with *Mbo*I overhangs were ligated to compatible adaptors and amplified according to the procedure described by Pires *et al.* (2004). The PCR products were cloned into the pDrive vector (Qiagen, Hilden, Germany). Plasmid inserts were screened by hybridization with the labelled genomic DNA. One clone that hybridized strongly with the genomic DNA was sequenced and designated CANR4 clone 4. No hits with GenBank were obtained after BLAST searches. The second sequence of CANR4 (clone 7) was obtained by PCR of *R. canina* genomic DNA using primers designed according to the consensus sequences of cluster 1 identified in whole genomic analysis using RepeatExplorer2 (see below). The sequences of the primers were as follows: Rcan_CL1_for: 5′-ATCTCACTAGAACAACGCA-3′ and Rcan_CL1_rev: 5′-GGGTTTAGGATTTGGTTTGG-3′. Amplification was performed using the following programme: denaturation at 92 °C for 3 min, 35 cycles of 92 °C for 20 s, 57 °C for 30 s and 70 °C for 30 s, and 5 min of extension at 70 °C. Sequences of Sanger clones were submitted to GenBank under accession numbers MK069592 [CANR4 clone 4 (CANR4.4)] and MK069593 [CANR4 clone 7 (CANR4.7)].

### Southern blot hybridization

The genomic DNAs were digested with restriction endonucleases (5 U µg^–1^ DNA, 6 h), fractionated by agarose gel electrophoresis and transferred to Hybond XL membranes (GE Healthcare, Marlborough, MD, USA) using alkaline capillary transfer. The probe was an insert of the CANR4.4 clone (GenBank MK069592) amplified with the universal vector Sp6 and T7 primers. Approximately 100 ng of purified PCR product was radioactively labelled with ^32^P-dCTP according to the manufacturer’s protocol (DecaLabel DNA Labelling Kit, Thermo Fisher, Waltham, MA, USA). Southern blot hybridization was performed in 0.25 m sodium phosphate buffer (pH 7.0) supplemented with 7 % (w/v) sodium dodecyl sulphate (SDS) at 65 °C. The membranes were washed with 2× SSC (10× SSC = 1.5 m NaCl, 0.15 m sodium citrate, pH 7.0), 0.1 % SDS (twice for 5 min) and then with 0.2× SSC and 0.1 % SDS (twice for 15 min at 65 °C). The membranes were exposed to a storage phosphor screen, scanned (Typhoon FLA7400; GE Healthcare), and the signal was quantified using Image Quant (GE Healthcare).

### High-throughput sequencing and sequence accessions

The sequencing project PRJNA380741 was supplemented with the newly sequenced genomes of *Rosa arvensis*, *R. majalis*, *R. spinosissima*, *R. canina* (Czech population), *R. rubiginosa*, *R. sherardii* and *R. corymbifera*. Data for *R. canina* (Germany), *R. inodora* and *R. dumalis* were taken from the project PRJEB15546 (Herklotz *et al*., 2018). Details for sampling and sequencing are provided in Table 1 and Supplementary Data Table S1, respectively.

### In silico identification of satellite repeats

The source read archives are listed in Table 1. The fastq reads were initially filtered for quality and trimmed to uniform length using the Pre-processing and QC tools in RepeatExplorer2 (Novák *et al.*, 2013). Read length ranged from 100 to 300 bp depending on the sequencing library and platform. The fastq>fasta conversion reads were analysed with the RepeatExplorer2 clustering program using default parameters. Typically, 100 000 paired-end reads or 200 000 single-end reads were used as inputs for RepeatExplorer2 clustering. This bioinformatic pipeline runs a graph-based clustering algorithm (Novák *et al.*, 2013) that assembles groups of frequently overlapping reads into clusters of reads, representing a repetitive element or part of a repetitive element with a higher-order genome structure. The similarity and structure-based repeat identification tools in RepeatExplorer2 aid in identification of the repeats. RepeatExplorer2 uses a BLAST threshold of 90 % similarity across 55 % of the read to assign reads to clusters (minimum overlap = 55, cluster threshold = 0.01 %, minimum overlap for assembly = 40), and the clusters are identified based on the principle of maximum modularity. The read archives outputs were searched for CANR4 sequences using a BLAST program implemented in ‘Utilities’. The copy number of rDNA units was calculated from the Illumina read count using the following procedure. (1) The genome proportion of CANR4 was calculated by subtraction of ‘non-CANR4 reads’ from the total reads in the cluster. (2) Calculation of genome space: genome proportion × size of the genome (Mb). (3) Copy number was then calculated: genome space values (bp) divided by the size of the unit (156 bp). Sequence homogeneity within each CANR4 cluster was estimated based on the total k-mer coverage score (ranging from ~0.4 to 0.6, a low-confidence satellite) reported by the Tandem Repeat Analyser (‘TAREAN’) tool (Novák *et al.*, 2017). The higher-order structure of cloned sequences was determined by dot plot analyses.

### Consensus sequence generation, alignment and phylogenetic reconstructions

We extracted consensus sequences from individual clusters using the TAREAN tool implemented within the RepeatExplorer2 program (Novák *et al.*, 2017). The consensus sequences were of different lengths and different frames (starting at different monomer unit positions) and matched opposite DNA strands. We dealt with these problems as follows. First, cluster consensus sequences were duplicated to make a tandem. Each duplicated sequence was aligned by the program BioEdit (Hall, 1999) using pairwise end-to-end alignment with the CANR4.4 monomer consensus. Both direct reverse and reverse complements were used. The aligned regions, ranging from 103 to 163 bp of each sequence, were extracted and copied to a separate file comprising ‘in-phase’ repeats. In a few cases for which TAREAN did not report a ‘satellite’, the corresponding consensus was obtained by mapping the reads to the CANR4.4 monomer consensus. Forty-seven sequences, including both Sanger clones, were subjected to multiple alignment using a tool in the CLC genomic program (parameters: 10_gap open cost, 1.0_gap extension cost, as any other end gap cost yielded a less accurate alignment mode). The multiple alignment was adjusted manually. The substitution model JC69 (Jukes and Cantor, 1969) fitted the data best according to the model tests performed by the program Mega v.6 (Tamura *et al.*, 2013). Based on this model, a maximum-likelihood algorithm implemented in the program SeaView v.4.7 (Galtier *et al*., 1996) was used to build a phylogenetic tree with an initial neighbour-joining tree and 1000 bootstrap replicates. In addition, a Bayesian phylogeny was calculated with MrBayes v.3.2.7a (Huelsenbeck and Ronquist, 2001; implemented in Geneious v.10.0.9) using default parameters (generation = 1.100.000; burn-in = 1.00.000)

### Single-nucleotide polymorphisms

CLC Genomics Workbench 11.1 (Qiagen) was used to estimate intragenomic variation between multiple CANR4 units. The frequency of single-nucleotide polymorphisms (SNPs) and genome proportion were determined using total Illumina reads trimmed for quality (Phred score ≥30 over ≥95 % of the read length). Trimmed reads (typically >7 million) were mapped to corresponding satellite monomers using the following parameters: insertion and deletion costs_3, lengths fraction_0.5, similarity fraction_0.8, deletion cost_2. The distribution of SNPs across the CANR4 sequences was recorded when the distribution exceeded a threshold of at least 20 identical SNPs over at least 200 reads that covered the variant position and occurred at ≥10 and ≥20 % frequency, respectively. The SNP frequency was expressed as the number of SNPs per 1 kb of DNA. The copy number was calculated according to the formula GP × GS × 10/monomer length, where GP is genome proportion (%), GS is genome size (Mb) and the monomer length is 0.158 kb. The 18S rRNA genes were more evenly covered than the other regions of rDNA and hence were preferentially used for copy number estimation.

### Fluorescence in situ hybridization

Mitotic metaphases were obtained from roots, pistils and anthers from young flower buds of *R. canina*, *R. inodora*, *R. dumalis*, *R. corymbifera*, *R. arvensis*, *R. majalis*, *R. spinosissima* and *R. gallica*. The material was sampled in the early morning and transported on ice water to the laboratory. For meiotic chromosome spreads, we used anthers from young flower buds ~0.5–0.8 cm long collected in spring. Flower buds were repeatedly collected during the period of flowering at 1-week intervals to obtain an optimal number of meiotic cells. The anthers were incubated in Triton X-100 for 5 min and washed in 2× SSC for 10 min; other pretreatments and fixations followed the protocol of Ma *et al.* (1996). Flower buds were incubated for 4 h at 25 °C in a solution of 0.008 m 8-hydroxyquinoline and 0.1 % colchicine and fixed in acetone/acetic acid (2 : 1) containing 2 % polyvinylpyrrolidone 40 000. Samples were conserved in 70 % ethanol and stored at −20 °C. Protoplasts were obtained by enzymatic treatment of tissue (0.5 h for pistils and 2 h for anthers) with 1 % cellulase, 0.2 % pectolyase Y23, 0.5 % hemicellulase and 0.5 % macerozyme R10 (Sigma-Aldrich, St. Louis, MO, USA) in citrate buffer (0.04 m citric acid and 0.06 m sodium citrate). Incubation in citrate buffer without enzymes was conducted for 1.5–4 h depending on the tissue.

FISH followed the procedures described by Lim *et al.* (2005) and Herklotz *et al.* (2018). Pistils and anthers were dissected and squashed on slides in a drop of 70 % acetic acid. The probes were amplified from plasmid clones using vector primers. The CANR4 probe was amplified from clone 4 (GenBank MK069592) using Sp6 and T7 universal primers; the 18S rDNA probe was a 1.7-kb fragment of the 18S nuclear rRNA gene of *Solanum lycopersicum* (GenBank X51576) amplified with the T3 and T7 universal primers. The probes were labelled by nick translation using Spectrum Green dUTPs (Abbott, Lake Bluff, IL, USA) for 18S rDNA and Cy3-dUTPs (Roche, Basel, Switzerland) for the CANR4 satellite. Slide preparation and hybridization were performed according to standard protocols (Schwarzacher and Heslop-Harrison, 2000). Chromosomes were counterstained with 1 μg mL^−1^ DAPI (4′,6-diamidine-2′-phenylindole dihydrochloride) diluted in mounting medium for fluorescence (Vectashield, Vector Laboratories, Peterborough, UK). The slides were scanned using epifluorescence microscopes (Olympus Provis AX70, Nikon ECLIPSE Ci) equipped with either an AxioCam MRm CCD (Zeiss, Oberkochen, Germany) or a Nikon DS-Qi2 camera (Nikon, Tokyo, Japan). The imaging software used was ISIS (MetaSystems, Altlußheim, Germany) and NIS-Elements BR (v.4.50, Nikon), respectively. All images were optimized for contrast and brightness using Adobe Photoshop v.11.0. Quantification of fluorescence intensity was conducted in TIFF images using Fiji software (Schindelin *et al.*, 2012). Regions of interest (probe hybridization sites) were selected using the drawing tools of the program. Integrated density per locus was counted in several meiotic cells of *R. canina*, *R. corymbifera*, *R. rubiginosa* and *R. inodora*.

## RESULTS

### Cloning and sequencing of a CANR4 satellite

To clone satellite repeats from *R. canina*, we employed a conventional strategy based on digestion of genomic DNA with a set of restriction enzymes and subsequent cloning of isolated fragments (details given in the Methods section). One genomic clone, CANR4.4 (GenBank MK069592), hybridized strongly with labelled *R. canina* genomic DNA on blots. Its insert was 595 bp in length, 64 % AT-rich and contained three tandemly arranged basic units, each ~160 bp in length (dot plot, e-value parameter < 0.001). The forward strand sequence aligned with the reverse strand sequence in several regions, indicating potential palindromic structure. A pattern search using a hidden Markov model revealed a dodecanucleotide motif, AT(c)TTCACTAA(g)AA, that was repeated nine times in the clone (three per 160-bp monomer). In addition, several dA_4–6_ tracks known to be centres of DNA curvature were identified. There were 339 purine and 256 pyrimidine residues, indicating considerable Pu/Py strand asymmetry, a feature that has also been reported in other satellites (Macas *et al.*, 2015). The second genomic clone, clone 7 (GenBank MK069593), was obtained by PCR amplification using the CANR4-specific primers. It was 548 bp in length and contained 3.4 copies of tandemly arranged 159-bp monomeric units. The two clones were 87 % identical, but clone 7 differed from clone 4 in that it contained an ~50-bp inverted duplication of the basic unit in each tandem. Only direct and no inverted repeats were identified in self-to-self comparisons of clone 7.

**Fig. 1.**
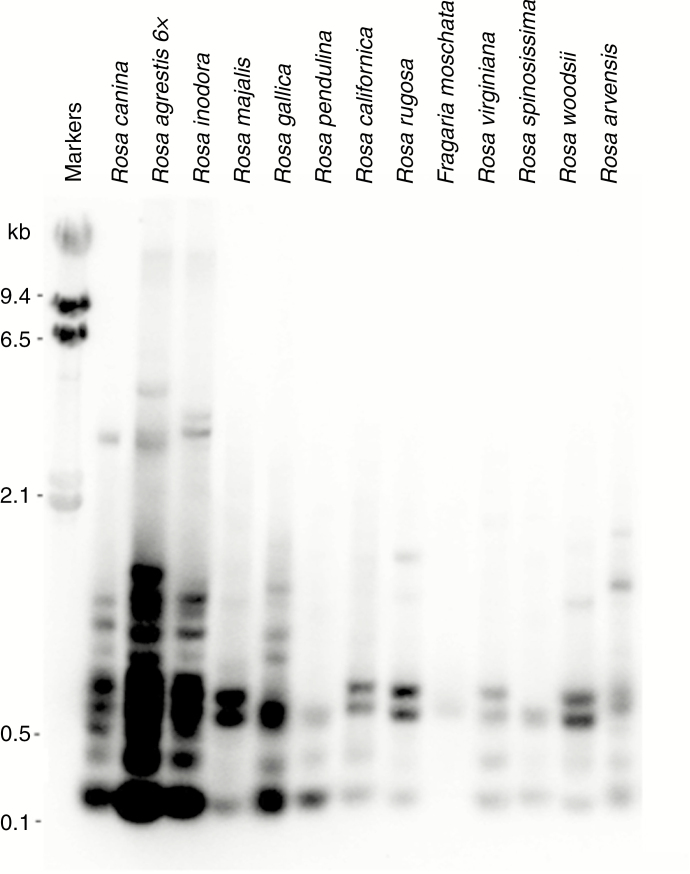
Southern blot hybridization of genomic DNAs digested with the *Mbo*I restriction enzyme. Note the strong signals in all three dogroses (5*x R. canina*, 5*x R. inodora* and 6*x R. agrestis*). Note the relatively strong hybridization to a 0.6 kb doublet of bands in most diploids.

The genomic organization and species specificity of the CANR4 repeats were further analysed by Southern blot hybridization. The *Mbo*I-digested genomic DNAs from several *Rosa* and one *Fragaria* species were hybridized with the CANR4 clone 4 (Fig. 1). The sizes of the hybridization restriction fragments ranged from ~150 bp to several kb. The ladders were often irregular, indicating multiple families of the satellite. Three dogrose species (5*x R. canina*, 5*x R. inodora* and 6*x R. agrestis*) showed stronger signals and more complex hybridization patterns than the diploid species. The probe did not hybridize with the DNA of *Fragaria moschata*.

### Identification of CANR4 in whole genomic sequence archives

**Fig. 2.**
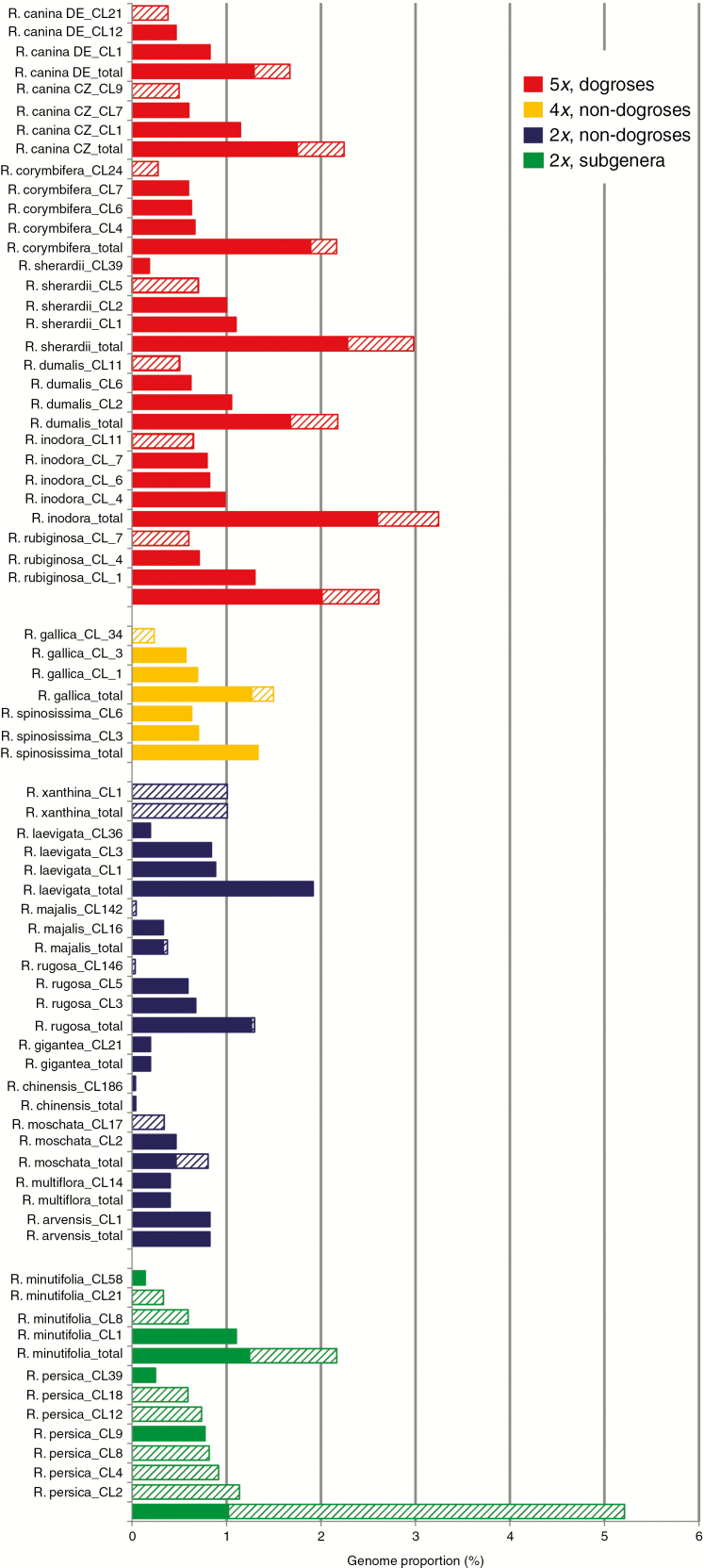
Abundance and diversity of CANR4-containing clusters. Columns indicate amplified CANR4 variants for each clade. Column height is proportional to genome representation. The total values are the sums of the genome proportions of individual clusters. Diploid, tetraploid and pentaploid dogroses are shown in blue, yellow and red, respectively. The diploid representatives of the two early divergent subgenera are shown in green. The consensus sequences of each cluster belong to two major clades in the phylogeny shown in Fig. 3. Clusters containing different CANR4 variants (Fig. 3) are indicated by filled (Clade 1) and hatched (Clade 2) columns.

**Fig. 3.**
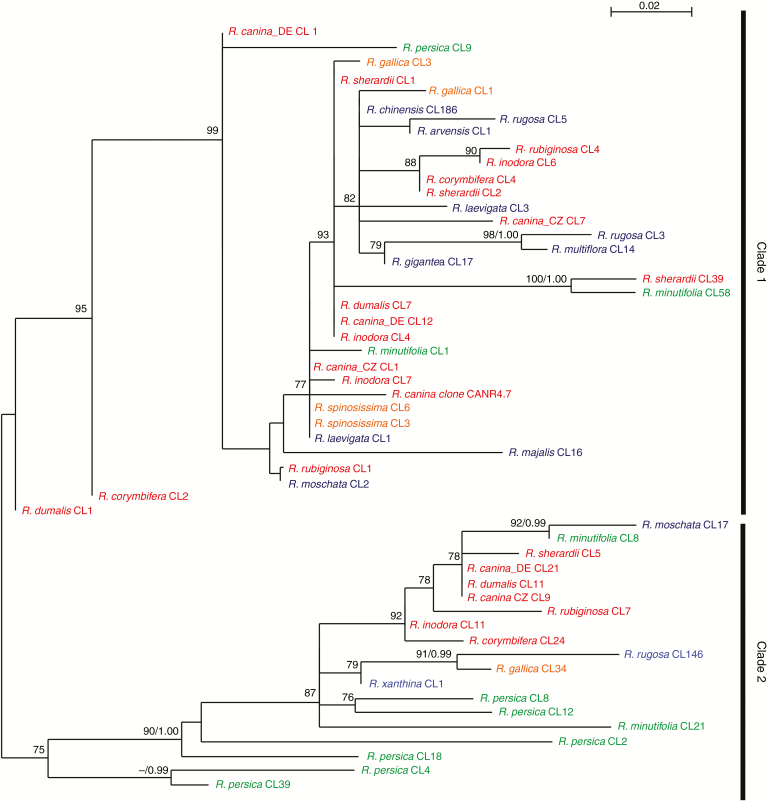
Maximum-likelihood (ML) phylogeny of consensus sequences generated from CANR4-containing clusters. Bootstrap values (BV>70 %) of the ML analysis and posterior probabilities (PP>0.95) of a Bayesian phylogeny were plotted on the 50 % majority rule consensus ML tree (BV/PP). Diploid, tetraploid and pentaploid dogroses are shown in blue, orange and red, respectively. The diploid representatives of the two early divergent subgenera are shown in green. The sequences are named according to the cluster number from which they derive. Low cluster numbers correspond to highly represented CANR4 variants (high genome proportion); high cluster numbers correspond to less abundant variants. The *R. canina* CANR4.7 clone (shown in red) groups within clade 1. Bootstrap support >70 % is indicated above the branches.

The previously described Southern hybridization blot experiment indicated the presence of a common satellite repeat in *Rosa*. To quantitatively analyse the satellite repeat, we used the available genomic archives listed in Table 1 and performed a clustering analysis using a RepeatExplorer2 pipeline for identification of the satellite in high-throughput reads (Novák *et al.*, 2017). Clustering was first run for each species separately to determine CANR4 satellite genome proportion and homogeneity (Fig. 2). The cumulative genome abundance (sum of the read abundance of individual clusters) was higher among dogroses (2.33 ± 0.23 %) than across non-dogrose species (1.31 ± 1.79 %; *t*-test: *t*_20_ = 5.41; *P* < 0.001, Supplementary Data Table S2). A wide range of genome proportions (0.04–5.21 %) was observed, particularly among the diploids. Dogroses contained three to four clusters, whereas one to three clusters were found in 2*x* and 4*x* roses. However, both diploid members of the basal subgenera *Hulthemia* and *Hesperhodos*, *R. persica* and *R. minutifolia*, respectively, contained four to seven clusters, and the cumulative abundance of CANR4 repeats was high. There was a relationship between satellite copy number and genome size (Pearson’s *r* = 0.81, *P* < 0.001; Supplementary Data Table S2), whereas the relationship between genome proportion and genome size was much weaker (Pearson’s *r* = 0.3, *P* < 0.001). Because we detected two major clades of CANR4 sequences across the genus *Rosa* (Fig. 3 and further below), most species contained clusters of both clades. The highest abundance of clade 1 clusters was found in dogroses, but clusters belonging to clade 2 were most frequent in *R. persica* (Fig. 2). The sequence read archives of *Fragaria moschata* (SRR5275222) and *Fragaria vesca* × F. viriginiana (SRR5381293) did not show any CANR4-related sequences in a BLAST search.

### CANR4 satellite variants in Rosa genomes and their phylogenetic relationships

To determine the phylogenetic relationships of the CANR4 sequences, we extracted consensus sequences from individual clusters (reported by TAREAN) of each species (Fig. 2). The consensus sequences were aligned, and a maximum-likelihood phylogeny was constructed (Fig. 3). The sequences clustered into two clades, of which clade 1 was supported by 99 % bootstrap support. The pairwise identities between clade 1 and 2 monomers averaged ~89 %. Visual inspection of the aligned sequences revealed several indels that discriminated between the two clades (Supplementary Data Fig. S1). Clade 1 was represented by sequences from the major clusters (indicated by low cluster numbers) of all dogroses, 2*x* and 4*x* species and minor clusters (indicated by high cluster numbers) of *R. minutifolia* and *R. persica*. Clade 2 contained consensus sequences from minor clusters of dogroses and 4*x* species but major clusters from the early-diverging species *R. persica* and *R. minutifolia*. Except for *R. majalis, R. moschata, R. rugosa* and *R. xanthina*, all of which were represented by minor clusters, no derived diploids were found in clade 2. Thus, dogroses maintain diverse satellite variants that arose early during the evolution of *Rosa*. Next, we investigated whether the clade 1 and clade 2 repeats differ in intragenomic homogeneity. To examine the intracluster homogeneity, we used a k-mer analysis in which the total score value is proportional to the number of interconnected nodes within the cluster (0, minimum; 1, maximum). The boxplot in Supplementary Data Fig. S2 shows higher homogeneity in clade 1 clusters than in clade 2 clusters (*t*_43_ = 2.738, *P* < 0.01).

### Intragenomic homogeneity of CANR4 determined by SNP analysis

**Fig. 4.**
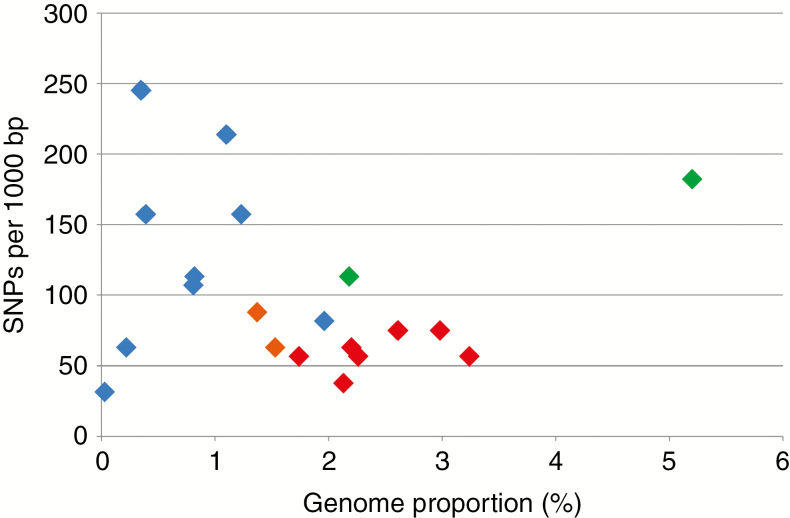
SNP frequencies and genome proportions of CANR4 satellites in 5*x*, 4*x* and 2*x Rosa* species. Pentaploid dogroses are indicated by red symbols, and tetraploid and diploid *Rosa* species are indicated by orange and blue symbols, respectively. The diploid representatives of the two early divergent subgenera are shown in green. Reads from individual clusters were mapped to the cluster consensus sequence and variants called at ≥20 % frequency. Increased sensitivity calls resulted in proportionally elevated SNP counts (Supplementary Data Table S2). On the *y*-axis, SNPs occurring in >20 % of reads; *x*-axis, genome proportion of the satellite.

The level of intragenomic homogeneity of the CANR4 satellite was estimated based on the number of SNPs in each cluster. Reads were mapped to the 158-bp overall CANR4 consensus sequence generated from the alignment (Supplementary Data Fig. S1) previously used to construct the phylogeny. SNPs were counted and plotted against the CANR4 genome proportion of the respective species (Fig. 4). Dogroses (red symbols) exhibited high intragenomic homogeneity as well as high abundance of CANR4. In contrast, most derived diploid species (blue symbols) contained very variable numbers of CANR4 sequences at a rather low abundance. The early-diverging diploid species of the genus, *R. persica* and *R. minutifolia* (green symbols), were clearly distinct in having high SNP frequencies and large amounts of satellite in their genomes. Dogroses had significantly fewer SNPs in CANR4 compared to diploid and tetraploid roses (*t*-test: *t*_20_ = −3.035; *P* < 0.01). Generally, there was no correlation between the genome proportion of CANR4 repeats and SNP frequency (Pearson’s *r* = −0.09, *P* < 0.001; Supplementary Data Table S2).

### Localization of the CANR4 satellite on mitotic chromosomes

**Fig. 5.**
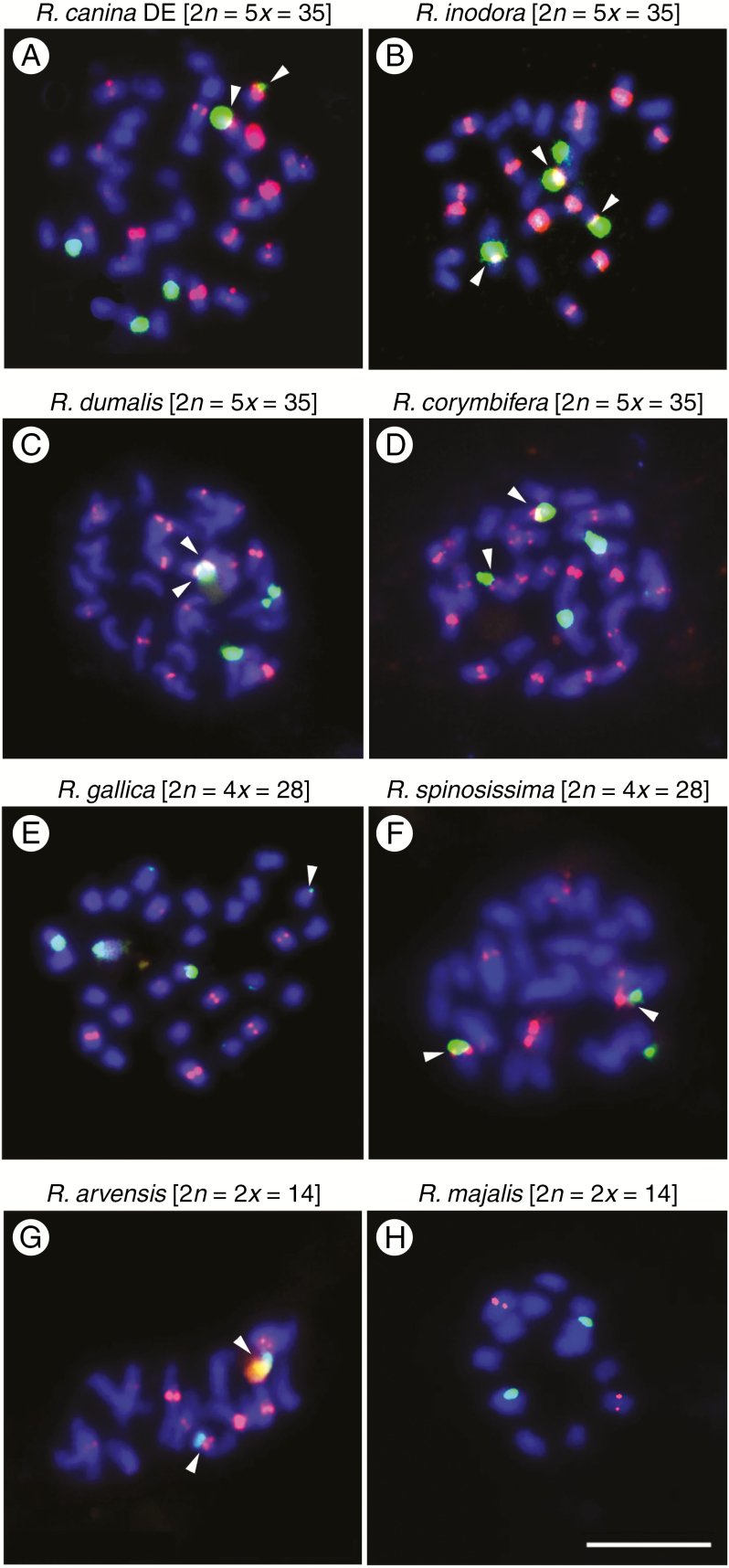
Mitotic metaphases of polyploid and diploid rose species after fluorescence *in situ* hybridization (FISH) using the CANR4 satellite repeat (red) and 18S rDNA (green). (A) *Rosa canina_DE* (5*x*, subsect. *Caninae*). Note the strong CANR4 signal on a minor NOR chromosome. (B) *Rosa inodora* (5*x*, sect. *Caninae* subsect. *Rubigineae*). (C) *Rosa dumalis* (5*x*, sect. *Caninae*). (D) *Rosa corymbifera* (5*x*, sect. *Caninae*). (E) *Rosa gallica* (4*x*, sect. *Gallicanae*). (F) *Rosa spinosissima* (4*x*, sect. *Pimpinellifoliae*). (G) *Rosa arvensis* (2*x*, sect. *Synstylae*). (H) *Rosa majalis* (2*x*, sect. *Rosa*). Arrowheads indicate co-localization of CANR4 signals and 18S signals on the same chromosome. Scale bar: 10 µm.

FISH was conducted to determine the number and position of CANR4 satellites on mitotic chromosomes (Fig. 5). We selected four dogrose 5*x* species (*Rosa canina*, *R. inodora*, *R. dumalis* and *R. corymbifera*, all section *Caninae*); non-dogrose species were represented by *R. arvensis* (*Synstylae*), *R. gallica* (*Gallicanae*), *R. majalis* (*Rosa*) and *R. spinosissima* (*Pimpinellifoliae*). The chromosomes were hybridized with the CANR4 probe (shown in red) and with an 18S rDNA probe (shown in green). *Rosa canina*, *R. inodora*, *R. dumalis* and *R. corymbifera* (Fig. 5A–D) showed 16–20 CANR4 signals at pericentromeric positions. The intensity of the signals varied, and approximately two-thirds of the signals were considered as strong. There were four strong 18S rDNA signals in each species. The fifth 18S site was either weak (*R. canina*, *R. corymbifera*, *R. dumalis*) or absent (*R. inodora*). The CANR4 sites co-localized with the 18S rDNA sites on two or three chromosomes. Except in the case of one *R. canina* chromosome, the satellite signals on NOR (nucleolar organizer region) chromosomes were generally weak. In 4*x R. gallica* (Fig. 5E), we detected 10–12 CANR4 sites of variable signal intensity. There were three major 18S rDNA loci and several minor 18S loci in *R. gallica*, consistent with a previous study (Fernandez-Romero *et al.*, 2001). The 4*x R. spinosissima* (Fig. 5F) had six to eight CANR4 sites, approximately four of which were strong. Three 18S rDNA sites were revealed in this species. The 2*x R. arvensis* (Fig. 5G) comprised eight to 11 CANR4 sites of moderate to weak intensity. The CANR4 probe hybridized to two sites in 2*x R. majalis* (Fig. 5H). The 18S rDNA probe hybridized to two sites in both *R. arvensis* and *R. majalis*.

### Distribution of the CANR4 satellite on meiotic chromosomes

**Fig. 6.**
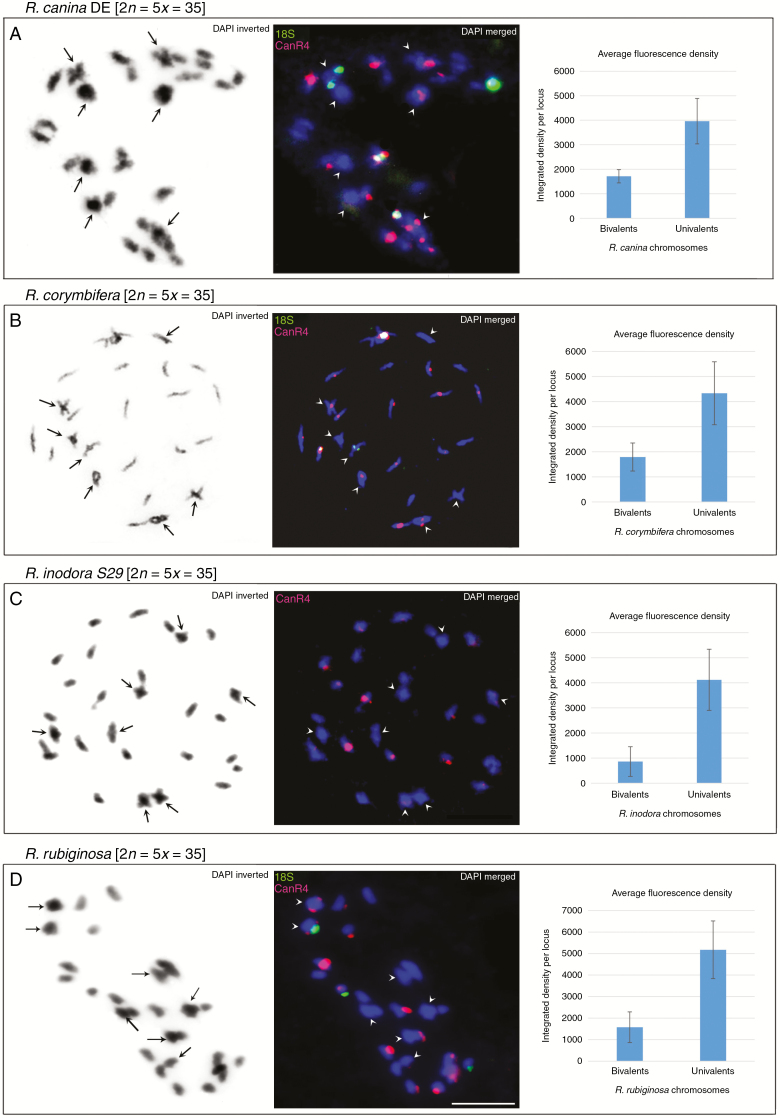
FISH of meiotic chromosomes. (A–D) Diakinesis of phase I shown for *R. canina_*DE (A), *R. corymbifera* (B), *R. inodora* (C) and *R. rubiginosa* (D). Left column: chromosomes stained with DAPI in greyscale. Arrowheads indicate bivalent chromosomes. Middle column: FISH of the same metaphase with the CANR4 (red) and 18S rDNA (green) probes. Bivalents are indicated by arrowheads. Scale bar: 10 µm. Right column: intensity of fluorescent CANR4 signals on univalent- and bivalent-forming chromosomes. The *y*-axis values represent the average signal intensity per locus; standard deviations were calculated from the data collected from four to 12 meiotic nuclei (Supplementary Data Table S3).

Meiotic pollen mother cells were analysed by FISH to study the distribution of the CANR4 satellite between bivalent- and univalent-forming chromosomes in 5*x* dogroses. We selected two representative species from each of the subsections *Caninae* and *Rubigineae*. Seven bivalent and 21 univalent chromosomes were identified at diakinesis in all four species (Fig. 6, left panels).

In *R. canina* (subsection *Caninae*), the CANR4 probe hybridized to two bivalents (four paired chromosomes) and to 12–16 univalent-forming chromosomes (Fig. 6A, middle panel). Two of the ten strong signals were located on bivalent (paired) chromosomes, and eight were located on univalents. Two univalent-forming NOR chromosomes carried both CANR4 and 18S signals, while none of the NOR bivalents showed co-localization of the two probes. The Czech sample of *R. canina* exhibited a distribution of CANR4 sites similar to that of the German sample, with strong signals on the univalent chromosomes (Supplementary Data Fig. S3).

*Rosa corymbifera* (subsection *Caninae*) had four strong and two weaker CANR4 signals on bivalents (Fig. 6B, middle panel). There were at least eight strong and several weak signals on univalents.

In the diakinesis of *R. inodora* (subsection *Rubigineae*), the CANR4 probe yielded four weak signals on bivalent-forming chromosomes and 11–15 mostly strong signals on univalent-forming chromosomes (Fig. 6C, middle panel).

In the diakinesis of *R. rubiginosa* (subsection *Rubigineae*), we detected three CANR4 signals on bivalents, one of which was co-localized with the 18S rDNA signal (Fig. 6D, middle panel) and one of which was generally very weak; the univalents carried eight strong and five or six weak CANR4 signals.

In each species, the number of CANR4 satellite loci and the repeat richness of loci (reflected by signal intensity) were statistically evaluated in four to 12 meiotic cells. The number of signals on bivalents was lower than the number of signals on univalent sets (Table 2, *P* < 0.001, Supplementary Data Table S3). To estimate the repeat richness of the loci, we quantified fluorescence signals on chromosomes (Fig. 6, right panels). It is evident that the average signal intensity per locus was higher on univalent-forming than on bivalent-forming chromosomes, and this was significant (*P* < 0.05) for all four species (Supplementary Data Table S3).

**Table 2.** Summary of FISH results showing the number and intensity of CANR4 signals

	Species, ploidy	Mitosis		Meiosis		
		Number of metaphases examined	Number of CANR4 sites^a^	Number of metaphases examined	Number of CANR4 sites^a^	
					Bivalents	Univalents
Dogroses	*R. canina*_DE, 5*x*	10	16–20	6	4x	12–16
	*R. canina*_CZ, ind. 1, 5*x*	8	17–18	10	4	13–14
	*R. canina*_CZ, ind. 2, 5*x*	–	–	10	4	12–15
	*R. canina*_CZ, ind. 3, 5*x*	–	–	8	4	12–14
	*R. corymbifera, *5*x*	10	19–20	4	6	11–13
	*R. dumalis, *5*x*	10	16–18	–	–	–
	*R. inodora, *5*x*	15	16–18	11	4	11–15
	*R. rubiginosa, *5*x*	–	–	12	6	12–13
Non-dogroses	*R. arvensis, *2*x*	10	8–11	–	–	–
	*R. gallica, *4*x*	10	10–12	–	–	–
	*R. majalis, 2x*	5	2	6	2	–
	*R. spinosissima, *4*x*	8	6–8	–	–	–

^a^Minimum to maximum fluorescent sites per metaphase. Metaphase to metaphase variation was mainly caused by small loci counts. Abbreviations: ind. = individual.

## DISCUSSION

Despite their peculiar behaviour in meiosis, dogrose chromosomes have only rarely been investigated using molecular cytogenetic approaches, and studies are limited to classical rDNA loci (Lim *et al.*, 2005; Khaitová *et al.*, 2014; Herklotz *et al.*, 2018). To gain insight into their structure and evolution, we isolated and analysed a pericentromeric satellite repeat that we named CANR4. This repeat has a wide occurrence across the genus, including the early divergent subgenera *Hesperhodos* and *Hulthemia*, suggesting its ancient origin close to the genus base estimated at *c*. 60 Mya (Xiang *et al.*, 2017; Zhang *et al.*, 2017). However, it was not found in *Fragaria*, a member of the sister clade to Rosaceae. Thus, CANR4 belongs to a class of genus-specific satellites with a relatively broad distribution involving subgenera, as reported in several plant groups (Hemleben *et al.*, 2007; Emadzade *et al.*, 2014; Belyayev *et al.*, 2018; Lee *et al.*, 2018). Even in species with low genome proportions (e.g. 0.03 % in *R. chinensis*), CANR4 represents a dominant satellite, and no other repeat seems to compensate for its relative deficiency. During the genome sequencing of *R. chinensis*, Hibrand Saint-Oyant *et al.* (2018) used an oligonucleotide probe (OBC226) to identify a pericentromeric satellite with low abundance. Although we did not explicitly analyse *R. chinensis* chromosomes, the similarity of hybridization patterns and the presence of the OBC226 oligonucleotide motif in both CANR4 clones suggest that CANR4 and OBC226 may be closely related satellites.

It is known that satellite variants may carry phylogenetic signals (Dodsworth *et al.*, 2015). Strikingly, despite the dramatic variation in copy number (up to 100-fold), the monomer sequence of CANR4 changed relatively little within the genus *Rosa*, suggesting low mutation frequency and slow evolution. Indeed, only two major groups could be distinguished in the phylogeny (Fig. 3). Of the rose diploids, only *R. xanthina* significantly amplified clade two variants. Interestingly, *R. xanthina* was placed as a sister species to *R. persica* in a whole-genome SNP phylogeny (Hibrand Saint-Oyant *et al.*, 2018), confirming the divergence of *R. xanthina* from the remaining rose species. In the genus *Prospero* (Hyacinthaceae), shifts in satellite abundance were correlated with dysploidy and cytotypic differentiation (Emadzade *et al.*, 2014). However, *Rosa* karyotypes are generally stable at euploid levels ranging from di- to heptaploid (Małecka and Popek, 1982, 1984; Ma *et al.*, 1997; Pachl, 2011; Kirov *et al.*, 2015; Herklotz *et al.*, 2018), suggesting that with the exception of NOR chromosomes (discussed below), alterations in repeat abundance are not associated with major karyotypic changes in this genus (Hibrand Saint-Oyant *et al.*, 2018). The two accessions of *R. canina* collected 300 km from each other differed in CANR4 content by as much as 25 % (Fig. 2). Given that the CANR4 satellite is present in high genome proportion, such variation may contribute to the genome size variability reported in Central European populations of *R. canina* (Ferus *et al.*, 2013). The location of the CANR4 satellite is strictly pericentromeric in both diploid and polyploid species, indicating its conserved chromosome position. It may be that the conserved chromosome position and the low sequence divergence of CANR4 contribute to chromosome synapsis of homologous chromosomes in interspecific hybrids, potentially influencing hybrid fertility (Lewis and Bayse, 1961).

### The CANR4 satellite is highly amplified in univalent chromosomes of 5x dogroses

Cytogenetic analysis of meiotic chromosomes showed that major CANR4 loci occur on univalent chromosomes, while bivalent chromosomes lack or contain fewer and minor CANR4 loci (Fig. 6). Thus, we are left with several possible explanations:

(1) One scenario could be that the bivalent chromosomes were donated by a progenitor with low CANR4 content. Conversely, the univalent chromosomes may originate from progenitor species rich in CANR4 repeats. Of note, the 2*x* synthetic *R.×ruga* Lindl., a hybrid between *R. arvensis* and *R. chinensis*, occasionally displays Canina-like meiosis in pollen mother cells (Wulff, 1954). Both parental species belong to the ‘*Synstylae* and allies’ clade, which also includes dogroses (Fougere-Danezan *et al.*, 2015). We demonstrated that *R. chinensis* has an extremely low genome proportion of the CANR4 satellite, while *R. arvensis* has a relatively high genome proportion and a high number of loci. Thus, evolution of pentaploid dogroses may have involved hybridization of species differing vastly in CANR4 content.

(2) Alternatively, the CANR4 satellite may have been amplified in pentaploid genomes after the hybridization event. This hypothesis is supported by several observations. First, all potential parental representatives from sections *Synstylae*, *Indicae*, *Gallicanae*, *Pimpinellifoliae* and *Rosa* (Ritz *et al.*, 2005) had lower content of the CANR4 satellite than any of the dogroses. Moreover, the FISH signals on univalent-forming chromosomes of dogroses were clearly larger than those on bivalents (Fig. 6). Second, a strong CANR4 signal was observed on a univalent-forming chromosome carrying a minor nucleolar organizer region (NOR chromosome) in *R. canina*, while NOR chromosomes generally harboured no or weak CANR4 sites in both tetraploids and diploids with regular meiosis (Fig. 5). Because there is a single NOR per monoploid set in *Rosa* (Ma *et al.*, 1997; Akasaka *et al.*, 2003; Kirov *et al.*, 2016), it is possible that one of the univalent-forming chromosomes underwent reconstruction in *R. canina*. Plastid and ribosomal DNA data suggest an independent origin of subsections *Caninae* and *Rubigineae*, respectively (Fougere-Danezan *et al.*, 2015; Herklotz *et al.*, 2018). We favour the hypothesis that the CANR4 satellite was amplified in univalent genomes independently, perhaps as a result of reduced recombination, which is known to eliminate repeats (Frank *et al.*, 2002). There is increasing evidence for the homogenization of tandem repeats, as witnessed here, in other systems with infrequent meiosis. For example, satellite DNA appears to be homogenous in apomictic *Hieracium* (Belyayev *et al.*, 2018). Furthermore, non-recombining univalent B chromosomes tend to accumulate satellites in *Prospero* (Jang *et al.*, 2013), rye (Klemme *et al.*, 2013) and other species (Marques *et al.*, 2018). Finally, 35S rDNA in univalent and bivalent chromosomes displays comparable homogeneity (Herklotz *et al.*, 2018), and there is no indication that univalents carry degenerate 5S rDNA families (Lim *et al.*, 2005). These case study observations suggest that some tandem repeats may not require frequent meiotic cycles for efficient homogenization but that they may instead rely on mitotic processes such as sister chromatid exchange (Henikoff *et al.*, 2001) and perhaps the activity of neighbouring transposable elements (Mestrovic *et al.*, 2015).

## CONCLUSIONS

According to Pele *et al.* (2018), polyploids tend to have more relaxed meiosis mechanisms and a higher rate of crossovers than diploids. Dogroses apparently escape this global trend, maintaining no or reduced recombination in univalent genomes over thousands of generations. There is increasing evidence that satellite DNA may have a function in chromosome pairing and segregation (Garrido-Ramos, 2017); thus, CANR4 (or the lack of large CANR4 loci) might be important for chromosome recognition. Expansion of satellite loci and perhaps other repeats may disrupt locus order, which in turn may lead to increased divergence between homologous chromosomes and the fixation of their univalent behaviour.

## SUPPLEMENTARY DATA

Supplementary data are available online at https://academic.oup.com/aob and consist of the following. Table S1: Details of genome skimming projects conducted within this study. Table S2: Genome proportion, copy and SNP numbers of CANR4 repeats calculated from high-throughput reads and statistical evaluation of the relationships. Table S3: Statistical evaluation of the number and intensity of FISH signals in bivalent- and univalent-forming chromosomes. Fig. S1: Alignment of CANR4 satellite consensus sequences from each cluster generated by the TAREAN program. Fig. S2: Intragenomic homogeneity of clade 1 and clade 2 satellite variants. Fig. S3: FISH analysis of additional meiotic nuclei of polyploid dogroses.

mcaa028_suppl_aob-20038-s01-s02-s06

mcaa028_suppl_aob-20038-s03

mcaa028_suppl_aob-20038-s04

mcaa028_suppl_aob-20038-s05
