# Supplementary material for: Asymmetrical canina meiosis is accompanied by the expansion of a pericentromeric satellite in non-recombining univalent chromosomes in the genus Rosa
Source: Ann Bot. 2020 Feb 25;125(7):1025–38. doi: 10.1093/aob/mcaa028 (PMC7262465; doi:10.1093/aob/mcaa028)
Supplement: mcaa028_suppl_aob-20038-s03 [file mcaa028_suppl_aob-20038-s03.pptx]

## Slide 1
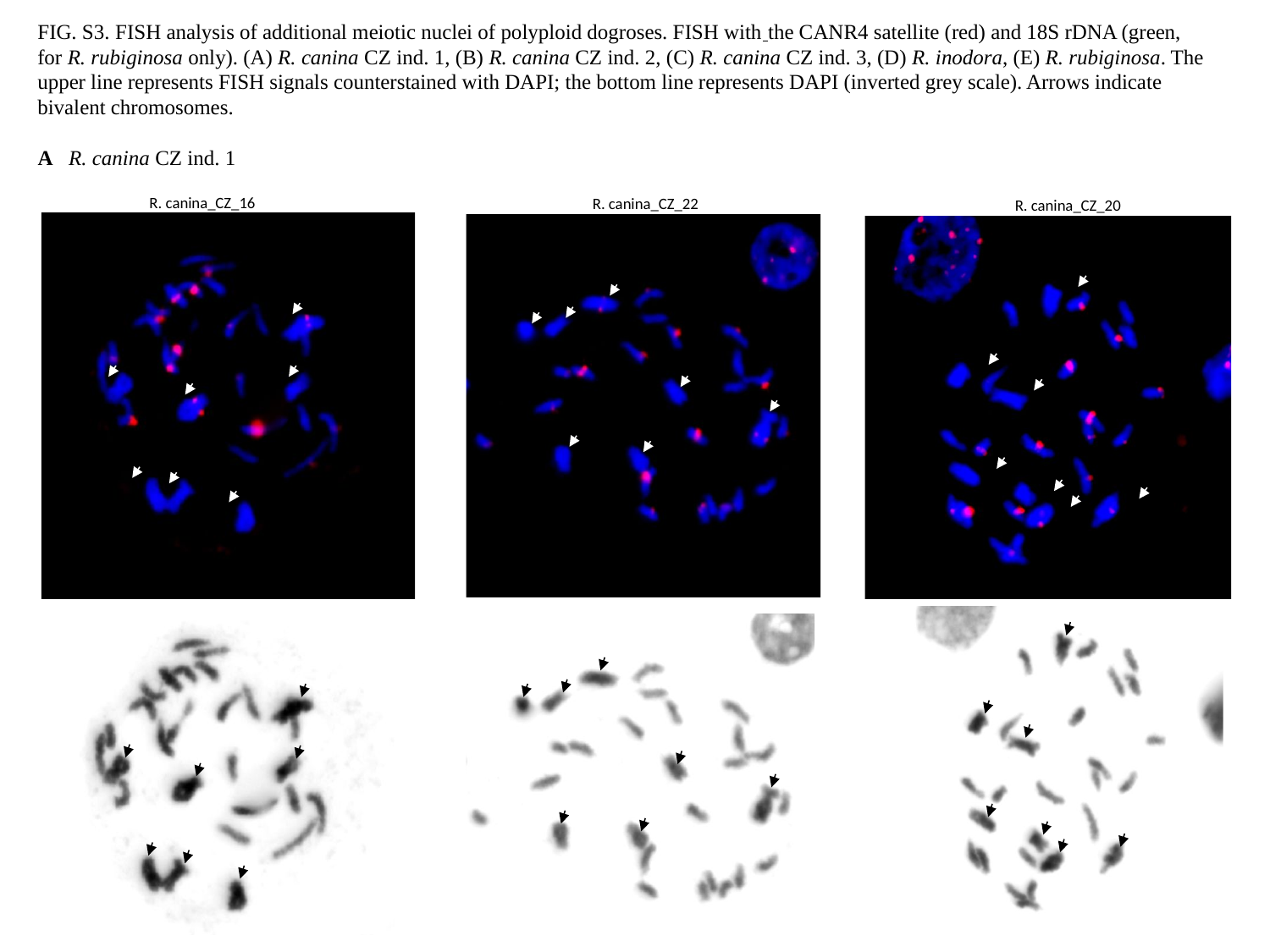

FIG. S3. FISH analysis of additional meiotic nuclei of polyploid dogroses. FISH with the CANR4 satellite (red) and 18S rDNA (green, for R. rubiginosa only). (A) R. canina CZ ind. 1, (B) R. canina CZ ind. 2, (C) R. canina CZ ind. 3, (D) R. inodora, (E) R. rubiginosa. The upper line represents FISH signals counterstained with DAPI; the bottom line represents DAPI (inverted grey scale). Arrows indicate bivalent chromosomes.
A R. canina CZ ind. 1
R. canina_CZ_16
R. canina_CZ_22
R. canina_CZ_20
1

## Slide 2
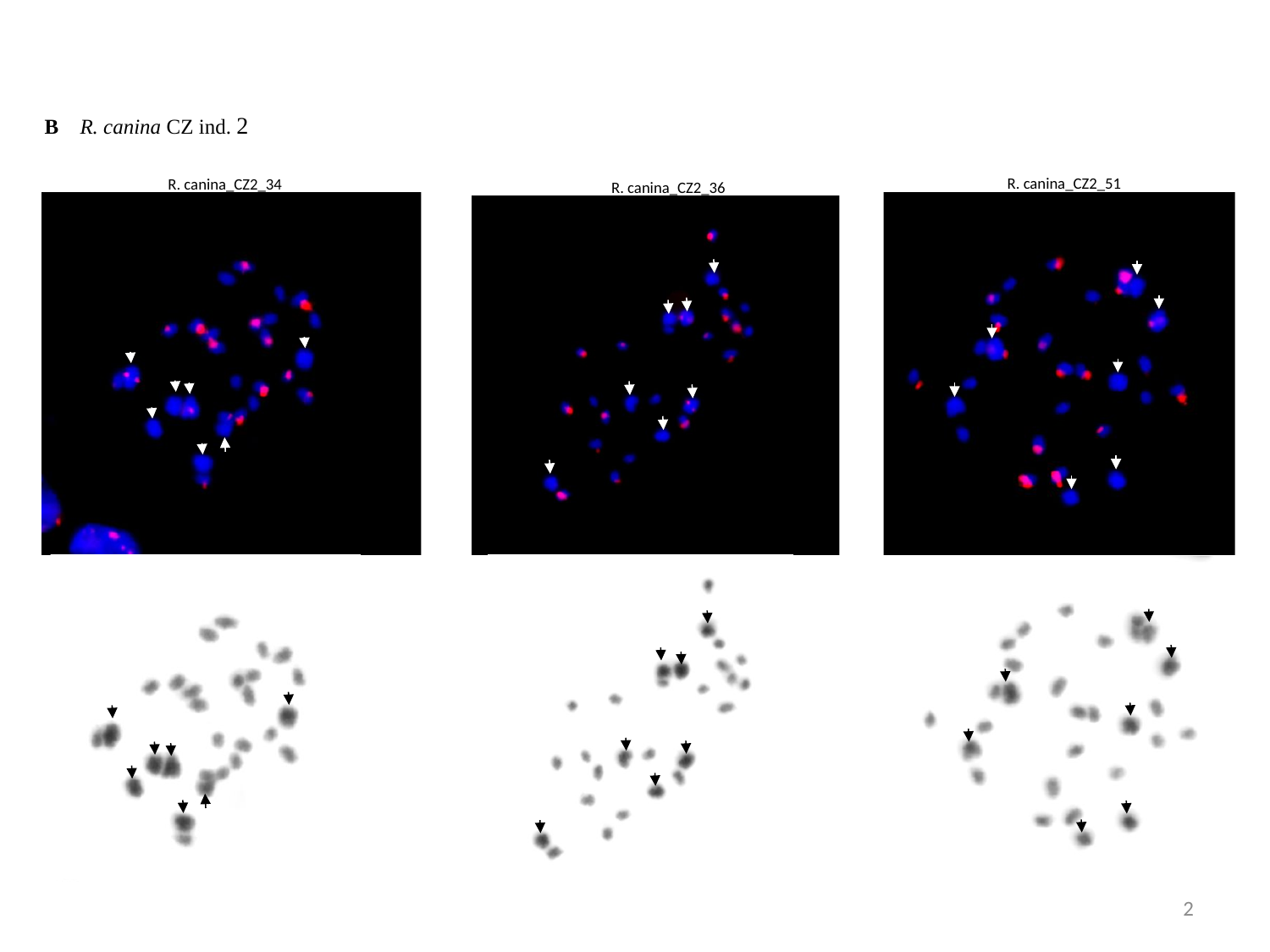

B R. canina CZ ind. 2
R. canina_CZ2_34
R. canina_CZ2_51
R. canina_CZ2_36
2

## Slide 3
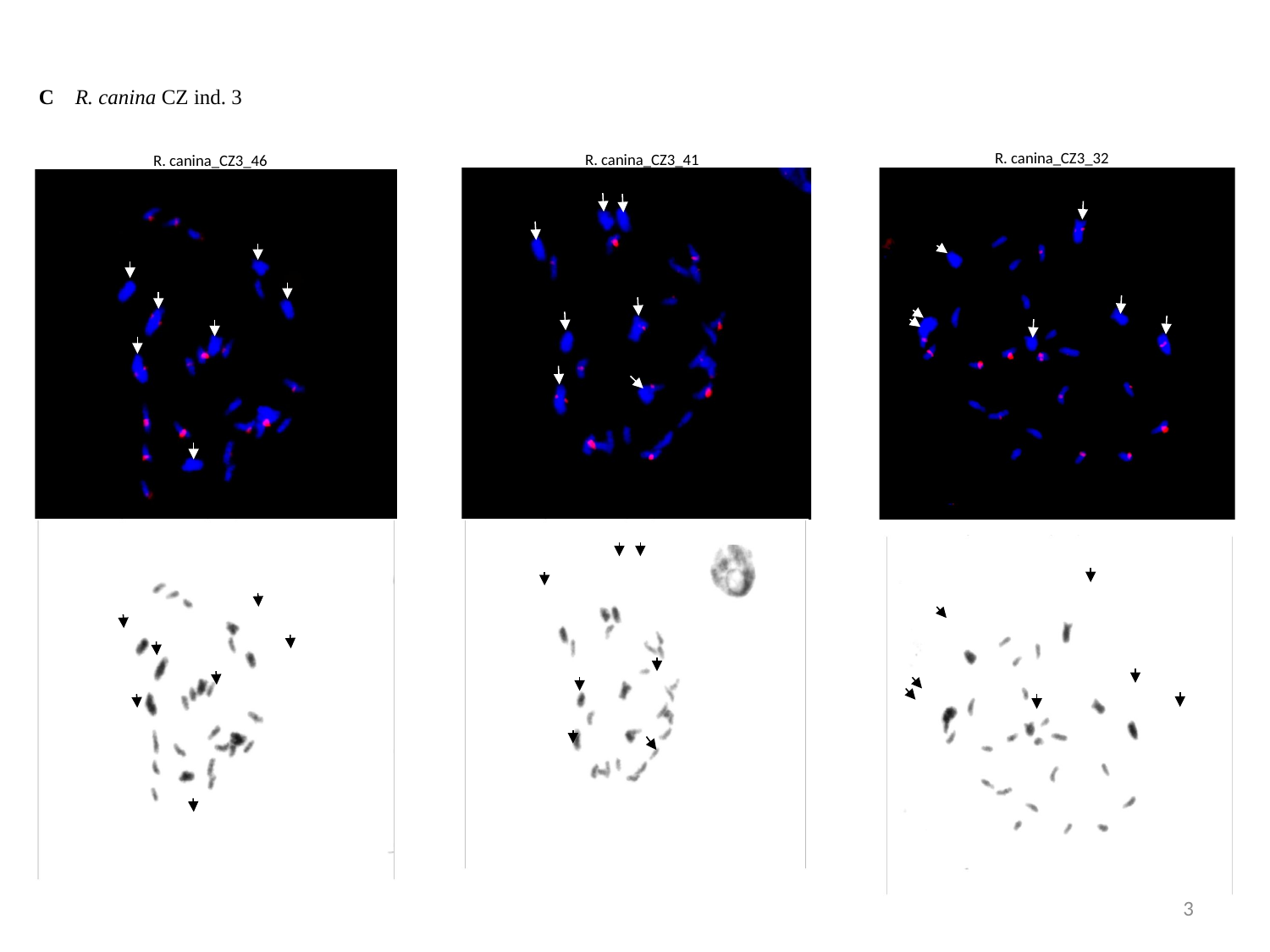

C R. canina CZ ind. 3
R. canina_CZ3_32
R. canina_CZ3_41
R. canina_CZ3_46
3

## Slide 4
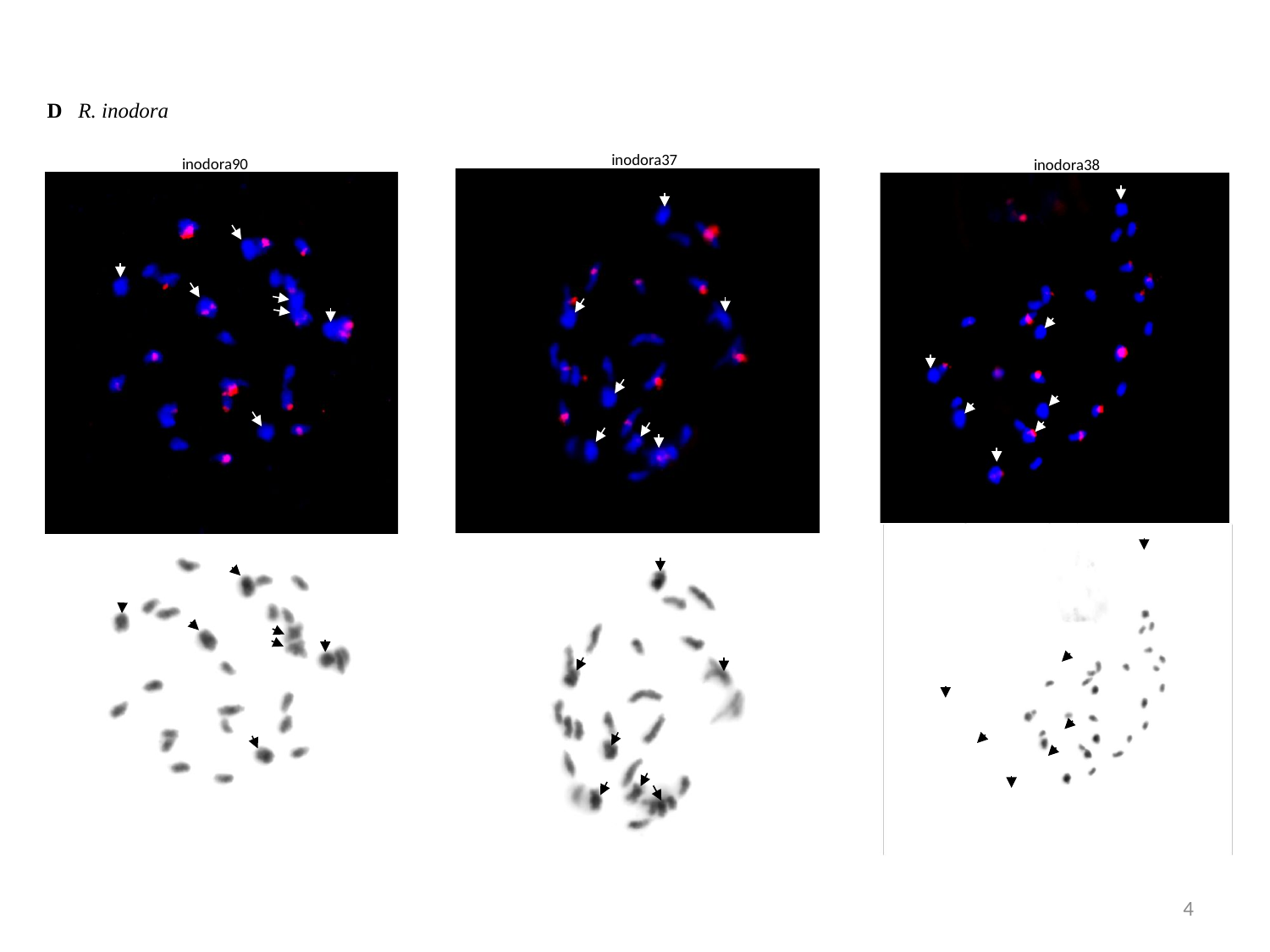

D R. inodora
inodora37
inodora90
inodora38
inodora38
4

## Slide 5
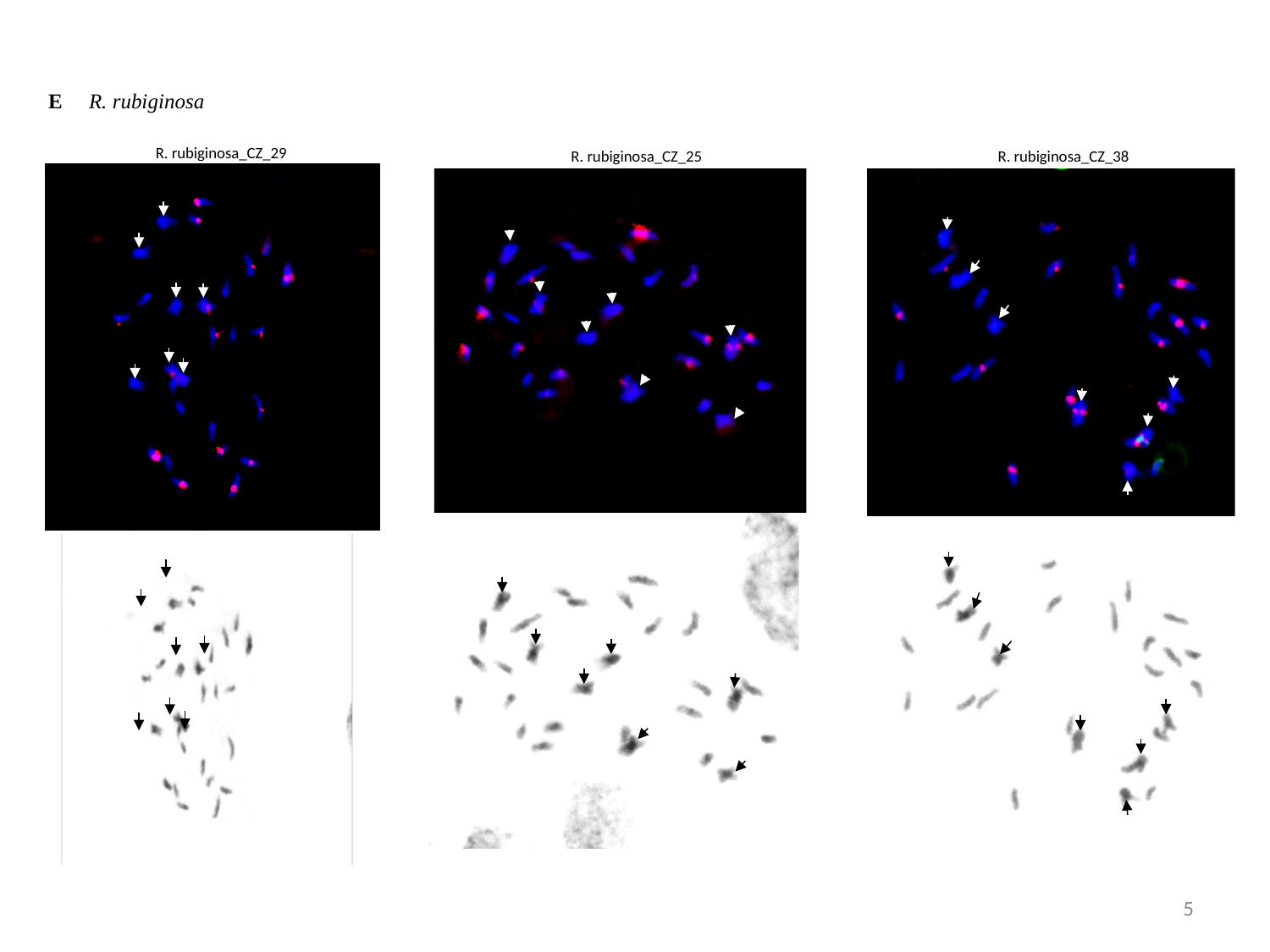

E R. rubiginosa
R. rubiginosa_CZ_29
R. rubiginosa_CZ_25
R. rubiginosa_CZ_38
5
