# Supplementary material for: Asymmetrical canina meiosis is accompanied by the expansion of a pericentromeric satellite in non-recombining univalent chromosomes in the genus Rosa
Source: Ann Bot. 2020 Feb 25;125(7):1025–38. doi: 10.1093/aob/mcaa028 (PMC7262465; doi:10.1093/aob/mcaa028)
Supplement: mcaa028_suppl_aob-20038-s04 [file mcaa028_suppl_aob-20038-s04.docx]

**Table S1.** Details of genome skimming projects conducted within this study

| Species | Sequencing platform/  company | Library | No. of pair end reads | Read length (bp) | Genome coverage^a^ | GenBank accession No. |
| --- | --- | --- | --- | --- | --- | --- |
| *R. corymbifera* | Illumina Hiseq4000/ BGI | Small fragment library, PCR free | 14,254,802 | 150 | ~1.5 x | SRR8265810 |
| *R. canina CZ* | Illumina Hiseq4000/ BGI | Small fragment library, PCR free | 14,237,076 | 150 | ~1.5 x | SRR8265808 |
| *R. rubiginosa* | Illumina Hiseq4000/ BGI | Small fragment library, PCR free | 13,854,388 | 150 | ~1,5 x | \| [SRR10402274](https://trace.ncbi.nlm.nih.gov/Traces/sra/?run=SRR10402274) \|  \| \| --- \| --- \| |
| *R. sherardii* | Illumina Hiseq4000/ BGI | Small fragment library, PCR free | 13,659,998 | 150 | ~1.5 x | [SRR10402273](https://trace.ncbi.nlm.nih.gov/Traces/sra/?run=SRR10402273) |
| *R. arvensis* | Illumina Hiseq4000/ BGI | Small fragment library, PCR free | 14,197,338 | 150 | ~3.9 x | SRR8265809 |
| *R. gallica* | Illumina MiSeq/FLI | TruSeq DNA nano | 1,159,206 | 300 | ~0.3 x | [SRR8422952](https://www.ncbi.nlm.nih.gov/Traces/sra/?run=SRR8422952) |
| *R. spinosissima* | Illumina MiSeq/FLI | TruSeq DNA nano | 3,846,970 | 300 | ~1.3 x | [SRR8422951](https://trace.ncbi.nlm.nih.gov/Traces/sra/?run=SRR8422951) |
| *R. majalis* | Illumina MiSeq/FLI | TruSeq DNA nano | 3,716,672 | 300 | ~2.3 x^b^ | [SRR8422953](https://www.ncbi.nlm.nih.gov/Traces/sra/?run=SRR8422953) |

^a^ data from the Plant DNA C-values Database (Bennett and Leitch, 2012). When no data were available average genome size of the 2x and 5x species was taken for calculations.

^b^ The genome size of the closely related *R. rugosa* was used in calculation because the genome size of *R.* *majalis* is unknown
