# Supplementary material for: Asymmetrical canina meiosis is accompanied by the expansion of a pericentromeric satellite in non-recombining univalent chromosomes in the genus Rosa
Source: Ann Bot. 2020 Feb 25;125(7):1025–38. doi: 10.1093/aob/mcaa028 (PMC7262465; doi:10.1093/aob/mcaa028)
Supplement: mcaa028_suppl_aob-20038-s05 [file mcaa028_suppl_aob-20038-s05.docx]

**Table S2**. Genome proportion, copy and SNP numbers of CANR4 repeats calculated from high-throughput reads

| **Species^a^** | **Genome proportion (%)** | **Genome size (Mb)** | **Copy number (rounded)** | **SNP20%/**  **1000 bp^b^** | **SNP10%/**  **1000 bp** |
| --- | --- | --- | --- | --- | --- |
| ***R. canina DE*** | 1.67 | 1418 | 150,000 | 57 | 107 |
| ***R. canina CZ1*** | 2.24 | 1418 | 201,000 | 63 | 164 |
| ***R.corymbifera*** | 2.17 | 1418 | 195,000 | 38 | 164 |
| ***R. inodora*** | 3.24 | 1394 | 286,000 | 57 | 170 |
| ***R. dumalis*** | 2.18 | 1379 | 190,000 | 57 | 113 |
| ***R. rubiginosa*** | 2.27 | 1394 | 179,000 | 44 | 145 |
| ***R. sherardii*** | 2.52 | 1394 | 200,000 | 38 | 145 |
| ***R. gallica*** | 1.50 | 1076 | 102,000 | 63 | 239 |
| ***R. spinosissima*** | 1.33 | 905 | 76,000 | 88 | 239 |
| ***R. rugosa*** | 1.30 | 489 | 40,000 | 157 | 270 |
| ***R. majalis*** | 0.37 | 719 | 17,000 | 245 | 277 |
| ***R. chinensis*** | 0.04 | 562 | 1,000 | 31 | 226 |
| ***R. gigantea*** | 0.19 | 548 | 7,000 | 63 | 151 |
| ***R. laevigata*** | 1.92 | 562 | 68,000 | 82 | 195 |
| ***R. xanthina*** | 1.01 | 391 | 25,000 | 214 | 403 |
| ***R. moschata*** | 0.80 | 581 | 29,000 | 107 | 314 |
| ***R. arvensis*** | 0.82 | 538 | 28,000 | 113 | 289 |
| ***R. multiflora*** | 0.40 | 807 | 20,000 | 157 | 245 |
| ***R. minutifolia*** | 2.17 | 557 | 76,000 | 113 | 264 |
| ***R. persica*** | 5.21 | 416 | 137,000 | 182 | 346 |

^a^ Dogroses – in red, non-dogrose polyploids – orange, Rosa diploids – blue, subgenera - green

**Statistical tests**

**T-tests:**

Comparison of genome proportion in dogrose and non dogrose genomes (t-test: t_20_ = 5.41;

P< 0.001)

Comparison of number of SNPs in dogrose and non dogrose diploid roses (t-test: t_18_ = -4.14; P<0.01)

Comparison of number of SNPs in dogrose and non dogrose genomes (t-test: t_20_ = -3.02; P<0.01)

**Pearson’s statistics:** Strength of association: 0 < | r | < 0.4 no significant correlation,

0.4 ≤ | r | < 0 .8 small correlation, 0.8 ≤ | r |≤ 1.0 strong correlation.

Relationship between genome size and CANR4 copy number, Pearson, r = 0.814

Relationship between genome size and CANR4 genome proportion, Pearson, r = 0.300

Relationship between genome proportion and SNP frequency, Pearson, r= -0.093

Relationship between SNP frequency and CANR4 copy number, Pearson, r = -0.506
